# Structural Determination of the Interaction of H_2_S and Insulin

**DOI:** 10.1063/4.0001121

**Published:** 2025-10-27

**Authors:** Christina S Rodriguez, Ming Fu, Rui Wang, Gerald F Audette

**Affiliations:** York University, Toronto, ON, Canada

## Abstract

Hydrogen sulfide (H_2_S) has emerged as an important biological signaling molecule. Its interaction with insulin impacts on glucose and lipid metabolisms. However, the molecular mechanisms underlying the cellular effects of H_2_S have been unsettled. To obtain direct evidence for H_2_S-induced post-translational modification of insulin molecule, we structurally characterized insulin following incubation with NaHS, an H_2_S salt, using X-ray crystallography. Insulin crystals were grown using the hanging drop vapor diffusion method and optimized in MES buffer with PEG MME550 and zinc sulfate. X-ray diffraction data were collected at the Canadian Light Source to resolutions between 2.1–2.2 Å. Six datasets were processed, structures solved and refined (representative structure PDB 9MRA; Rwork/Rfree 0.18/0.23). Structural analysis showed electron density corresponding to a sulfur atom near the amide group of Gln4 in chain B, aligning with prior LC-MS predictions. However, N–S distances ranged from 2.35–3.4 Å across the structures, suggesting the possibility of a transient covalent interaction disrupted by radiation damage during data collection. In addition, one structure exhibited a sulfur atom near Glu residues, suggesting secondary or alternative binding sites. All refined structures were predicted to form hexameric assemblies based on PISA analysis. These results provide structural support to H_2_S-induced post-translational modification of the insulin molecule.

